# Treatment of Erysipelas

**Published:** 1882-07

**Authors:** G. Frank Lydston

**Affiliations:** Late Resident Surgeon Charity and State Emigration Hospitals, New York. Lecturer on Genito-Urinary Diseases, College of Physicians and Surgeons, Chicago; 137 Madison St., Chicago


CHICAGO MEDICAL

JOURNAL & EXAMINER.

VOL. XLV.—JULY, 1882.—NO. 1.

### Original Communications.

ARTICLE I.

THE TREATMENT OF ERYSIPELAS. G. FRANK LYDSTON, M.D., Late Resident Surgeon Charity and State Emigration Hospitals, New York. Lecturer on Genito-Urinary Diseases, College of Physicians and Surgeons, Chicago. (Read before the Chicago Pathological Society, June 26, 1882.)

There are few diseases which have called forth a greater variety of methods of treatment in times past than erysipelas, and there are few diseases the management of which is at the present time of a more routine nature.

Since the introduction of the muriated tincture of iron into its treatment, and its high recommendation as a specific for the affection, it has been prescribed by physicians almost universally as a matter of routine practice.

The reputation it has gained has, however, been sustained by experience. The local measures which have been recommended, and which are still in favor with different practitioners, are greatly varied as the taste of the individual may select. The application of cold has been used with especial frequency, and applications of nitrate of silver, iodine, and various dusting powders, have had, and still have, their advocates.

There has been a tendency to either one of two extremes in the treatment of erysipelas, viz.: to keep in view its constitutional character, and to depend mainly upon the so-called specifics; or to consider the disease as merely of an inflammatory nature, and to treat it as a local inflammation. There is really little to offer that is new in the treatment of erysipelas, but as a large number of cases, especially of the severer types of the disease, do not usually come to the notice of the general practitioner, and are seldom seen under circumstances favorable for comparison of the results of different remedial measures, excepting by the surgeons of the larger hospitals, a general survey of the treatment found to be most useful in an observation of several hundred cases, may be of service. I shall endeavor to say but little aside from the treatment of the disease, merely mentioning a few of the interesting or practical points which I have had the opportunity of observing.

Erysipelas is usually classified as, first, simple cutaneous; second, cellulo-cutaneous, or œdematous (Lawrence); and third, cellular or malignant, the latter being also termed "diffuse cellulitis." The principles which govern the treatment of these different forms are essentially the same in all, and the classification is itself somewhat arbitrary, as is evident to any one who has observed the affection to any extent; for many cases of cutaneous erysipelas, particularly of the face, are accompanied by a certain amount of cellulo-cutaneous inflammation and suppuration; often with considerable sloughing of the tissues. This is seen especially in the tendency to suppuration and sloughing of the eyelids, and to the formation and burrowing of pus beneath the scalp in cutaneous erysipelas of the head and face. We frequently, also, see cases of the cellulo-cutaneous form which are essentially of a malignant type from their commencement. Cellulo-cutaneous erysipelas of the genital region, for example, has a special tendency to malignancy.

The first consideration in regard to the management of erysipelas, is evidently the question of prophylaxis.

Erysipelas is undoubtedly contagious, and is by some authorities regarded as extremely so. It appears to me, however, that its contagiousness is somewhat overestimated. Having had a large general surgical service, and a considerable number of cases of erysipelas under my charge at the same time, I have enjoyed an excellent opportunity of observing the contagious properties of the disease. The cases of erysipelas which developed primarily in the surgical wards were very few, but cases were from time to time accidentally introduced into the wards, through mistakes in the distribution of the patients. Such cases were, of course, immediately isolated, but free communication, through the medium of the assistants and nurses, was maintained with the erysipelas wards, no extraordinary precautions being taken to prevent contagion, although ordinary cleanliness was observed. I have also frequently seen patients with open wounds or ulcers exposed accidentally to contagion by being sent to the erysipelas pavilions by mistake, without their contracting the disease.

During a period of eighteen months there was no epidemic of erysipelas in the hospital, nearly all cases having developed in the city previous to their entrance into that institution. Caution, however, should be exercised, in spite of the facts mentioned, inasmuch as a considerable degree of contagiousness undoubtedly exists. This fact indicates the measures of prophylaxis. Strict cleanliness on the part of attendants, and in the care of surgical instruments which may be brought in contact with the case, with immediate and careful isolation of the affected, are to be strongly insisted upon. The caution necessary to prevent the transmission of contagion to the lying-in room is too obvious to require comment. As conditions of debility predispose to erysipelas, the systems of persons exposed to contagion should be maintained above par, if possible; particularly in prospective operative cases.

We now come to the general considerations governing the curative treatment of the disease.

In all cases, of whatever type, the asthenic character of the inflammation must be borne in mind, and should constitute the chief indication to be considered, our chief aim being to prevent or counteract exhaustion. This bears more especially upon the cellulo-cutaneous and cellular forms. The presence of any constitutional fault or cachexia, or of chronic alcoholism, renders the tendency to the typhoid condition still more marked, and should be combated accordingly. The danger of suppuration and sloughing, which perhaps may be followed by septic infection, with its train of attendant evils, must also be remembered.

The constitutional condition depends largely upon the extent and intensity of the local inflammation, thus enhancing the importance of local treatment, and the selection of such remedial applications as will give the greatest amount of relief.

In the treatment of simple cutaneous erysipelas in a healthy person, whose system is not debilitated by chronic alcoholism or privation, and who is free from any cachectic condition, and in whom there is no complication, but little is required save attention to hygiene and measures to allay local irritation and fever; these cases tending to recovery even without interference, although comparatively slight influences may render them serious.

It had been my practice to administer the tincture of the chloride of iron with chlorate of potassium in large doses, preceding it by a brisk saline cathartic, and using as a local application lead and opium in cold solution, with attention to tonic and supporting measures as required.

The usual hospital routine varied but little from this; and it is, I believe, the method of treatment resorted to by a great many practitioners. In a certain proportion of cases suppuration of greater or less extent ensued; and in view of the fact that the continuous application of cold to a tissue affected with a low type of inflammation still further lowers its vitality, thus favoring the formation of pus, I began the use of moist heat, continuously applied, covering the affected part with sheet lint, saturated with a solution of lead and opium as hot as could be borne, this being renewed as frequently as necessary to maintain an even degree of heat. I also combined salicylic acid with the iron, instead of the chlorate of potassium, with the idea of obtaining the antiseptic and antipyretic effect of the former drug, having in view the specific and constitutional character of the disease. The results were extremely gratifying, the hot applications rapidly reducing the swelling and tension of the part, with marked relief of the pain, which rendered them especially grateful to the patient. By thus affording a greater amount of relief to the local irritation, much more marked reduction of the constitutional disturbance was noticed than had previously been obtained by the application of cold. The combination of the salicylic acid with the iron acted remarkably well, the duration of the cases being shortened, and the fever and tendency to asthenia diminished. Subsequently, in the *London Practitioner* for October, 1879, I observed a formula for the preparation of the salicylate of iron, published by Walls White, which I have found to be a still better preparation for the administration of the two drugs in combination. With some modifications, it is as follows:

| | | |
|---|---|---|
| ℞. | Ferri sulphat .................................... | ʒi ℈ii. |
| | Sodii salicylat.................................... | ʒii. |
| | Sodii acetat...................................... | ʒi ℈i. |
| | Glycerinæ ...................................... | ℥iv. |

M. Sig. ℥ss dose.

Each ounce of this preparation is said to contain thirty grains of the salicylate of iron, the dose being found ʒii to ℥ss, which may be given every two to four hours, according to the exigencies of the case. I have found this preparation to be all that was claimed for it. It is less liable than the tincture of the chloride of iron to produce gastric irritation or constipation; it promotes diaphoresis, and has a decided effect upon the temperature and pulse. I have used the same drug after surgical operations to prevent septicæmia, in puerperal septicæmia, and in many febrile affections as a substitute for quinia, with marked benefit. The excessive doses of the tincture of the chloride of iron so often administered are, I think, injurious, causing disturbance of both the stomach and bowels; and, moreover, irrational, inasmuch as the absorption of such large quantities is hardly probable, on account of the irritation and functional disturbance of the gastric mucous membrane which is produced by them.

In cases of cutaneous erysipelas which are decidedly sthenic from the beginning, or in the severer forms of the disease under similar circumstances, the old-fashioned combination of calomel and opium, still clung to tenaciously by many surgeons, more especially in England, is undoubtedly advantageous, and it is my own custom to administer it for the first twenty-four or thirty-six hours after the onset of the disease, in such instances. It seems to modify and shorten the course of the inflammation, limiting exudation, and the tendency to the formation of pus. We also obtain the anodyne effect of the opium. It should be used only in the sthenic cases, and then with caution, keeping in mind its depressant effect, and the danger of ptyalism. I ordinarily give about gr. j of the mercurial, with from gr. $\frac{1}{2}$ to gr. j of opium every two or three hours.

In small children, the simple form of erysipelas is probably best treated by painting the affected parts with tr. benzoin co. or liq. ferri persulph., and then swathing them in cotton wool, with the adminstration of good food and small doses of quinine and iron. I have seen a number of such cases of simple cutaneous erysipelas in young children confined in a hospital ward, apparently as the result of damp. The ward was one used for convalescent paupers, and as a nursery. For some time, occasional outbreaks of cutaneous erysipelas in the children, and of acute articular rheumatism among the women, had occurred, and upon investigation, these had been found to be coincident with the occasional scrubbings which the floor of the ward received, it being imperfectly dried thereafter. As soon as this state of affairs was properly regulated, the erysipelas and rheumatism no longer occurred. The children recovered, with but one exception, although the erysipelatous blush covered over nearly the whole body in several instances. The case that proved fatal was one in which the navel had been neglected by the midwife, and in which gangrene of the abdominal walls occurred.

The application of the tincture of iodine, and the attempt at limitation of erysipelas by the use of nitrate of silver, are not only inefficacious in accomplishing the object sought, but appear to be unnecessary sources of irritation in the majority of instances. I have given them both a fair and extended trial. Cold applications of the muriated tincture of iron, of salicylic acid, or of the compound tincture of benzoin, are undoubtedly beneficial, but in no wise to be compared with moist heat, especially when it is combined with a sedative. Especial attention should be paid to these applications, for if they are allowed to become cold, they will prove injurious. Where they cannot be faithfully and continuously applied, cold applications of some of the drugs mentioned will prove more reliable. It is sometimes well to combine a diaphoretic with the internal remedies. In cutaneous erysipelas of the face, the parts should be covered with a mask of sheet lint, dipped in the hot solution of lead and opium. The ordinary lead and opium lotion in use in the New York hospitals contains about ʒii of the acetate of lead, with ℥ss of the tincture of opium to the pint of water. My own practice is to prepare a solution of about double this strength, thus increasing its anodyne and sedative effects. A saturated solution of the bicarbonate of soda, so highly extolled in the treatment of burns, is also an excellent local application.

Especial attention should be given to the condition of the eyelids, and if they are very tense and swollen, or appear boggy and infiltrated, with a great probability of suppuration or sloughing, free incisions ought to be made at once. These incisions should be made in the long diameter of the lid, and sufficiently free to allow the escape of the serous fluid with which they will be found to be infiltrated. Little or no scarring results, although the incisions may seem quite extensive, on account of the extreme swelling of the parts. It is better to make early incisions in this manner, even though the probability of sloughing be not very great, than to run any risk of its occurrence. Should sloughing supervene, great deformity and cicatricial contraction of the lids may result, and prove a serious calamity. The scalp should be very carefully watched, as suppuration and burrowing beneath it may occur very insidiously, resulting in troublesome sickness, if not in destruction of the pericranium and caries of the bones of the skull. Should any pocketing of pus be detected, free exit should be given to it at once.

There is often, in extensive facial erysipelas, severe conjunctivitis with profuse secretion. In such an event, the secretions should be carefully removed, and the inflammation allayed by frequent and prolonged syringing with warm water, containing about ʒii of the bicarbonate of soda to the pint. Erysipelas of the head, neck, and face, either singly or collectively, is quite liable to be accompanied by considerable cerebral disturbance, which calls for some modification of the treatment. These cases may be dependent upon erysipelatous inflammation of the cerebral structure or its membrane, or may occur independently of it, no lesion whatever being found explanatory of the symptoms. I have seen in several instances, notably in a case of cellulitis of the neck, and another of erysipelas of the orbit, symptoms which seemed decidedly to indicate meningitis, but in which, upon autopsy, no pathological evidence of cerebral or meningeal inflammation were detected. In those cases involving the neck, disturbance of the return circulation from the brain has been given as an explanation of the symptoms; but as the same symptoms, and in a more marked degree, are seen in case of erysipelas limited to the orbit, or to situations remote from the veins returning the blood from the cranial cavity, and no marked evidences of cerebral congestion are found, this view is hardly tenable. I think that there is some peculiar effect produced upon the cerebral substance by the erysipelatous poison, which gives rise to its profound functional disturbance, independently of inflammatory changes, or conditions of congestion, in a manner analogous to the action of other toxic poisons which produce marked effects upon the functions of the brain. Such cases as those I have just described are not, of course, to be classed under the head of cutaneous erysipelas, but as the cerebral symptoms produced by the latter are under consideration, they would seem to be appropriately discussed in that connection.

If the delirium be of the active variety, as is probable if the erysipelas be sthenic in character, a brisk saline cathartic should be given, the head shaved, and an ice-cap applied. Internally, a combination of bromide of sodium, ergot, and chloral hydrate will be found most beneficial. Should the temperature be high, cold sponging or the wet pack should be resorted to. The cold bath is recommended by Volkman, but seems rather too radical a measure for such cases. Ringer gives aconite, and Lobbe advises veratrum viride. Either of these drugs will prove useful, but must be cautiously given. If the delirium be of the low, asthenic type, measures to control high temperature should be conjoined with free stimulation, and the administration of opium, the latter being best given as the improved Dover's powder, containing the bromide instead of the sulphate of potassium.

Delirium is especially liable to occur if the patient has been addicted to the use of alcohol, and its treatment should be conducted upon essentially the same principles as in other cases. The cases which are due to extension of the inflammation to the cerebral structures, or to the condition simulating it, are to be treated as suggested for the cases of simple delirium, but with little hope of success, as they are almost invariably fatal. In addition to the ice-cap and other measures, leeches should be applied to the mastoid region. In the milder cases of delirium, this is hardly advisable, as there is some danger of the leech bites acting as new foci for the inflammation.

Erysipelas of the fauces may occur in erysipelas of the head and face, and may lead to œdema glottidis, or to the severer forms of inflammation and sloughing. The deeper seated varieties of erysipelas of the neck are especially liable to extension to the fauces, with resulting œdema of the glottis. Erysipelas of the fauces may, however, occur primarily, and remain circumscribed. In the treatment of this complication, the application of the nitrate of silver in the strength of forty grains to the ounce, if applied at the outset of the inflammation, will prove useful, and frequently abort it. Gargles of sulphurous acid in hot solution, with the same remedy internally, are useful. Small pieces of ice, instead of the hot gargle, may be given. Hot poultices should be applied to the neck. If convenient, steam inhalations will be excellent, and very grateful to the patient.

The effect of erysipelas upon chronic skin troubles may not be out of place if mentioned in connection with cutaneous erysipelas. I recall a case of the form of leprosy, known as elephantiasis Græcorum, in which cutaneous erysipelas supervened. It was pronounced "leprosy fever" by an enthusiastic dermatologist, who predicted a fresh eruption of tubercle. The case proved to be erysipelas, however, and was followed by a marked improvement. The acute inflammation evidently excited a new action, resulting in resolution and absorption of the infiltration to a certain extent. About as fortunate an accident as can occur in a chronic ulcer or eczema of the leg, is the supervention of erysipelas, provided the patient be in good condition, and the inflammation not too extensive. On the subsidence of the acute symptoms, resolution of the indurated tissue about the ulcer, and rapid healing frequently ensue from the new action thus induced. As regards the relation of syphilis to erysipelas, I have seen some cases in which the inflammation seemed to determine the eruption as a result of general systemic disturbance, and again, have seen an eruption of syphilis fade with unusual rapidity upon the occurrence of erysipelas.

It is always well, however, in any case of erysipelas in which there is a syphilitic history, to administer a mercurial, especially during the healing of any ulcers or sinuses which may have resulted.

The cellulo-cutaneous or phlegmonous erysipelas is in all instances a serious affection, and both the constitutional and local measures are proportionately important. The marked tendency to asthenia, and the suppuration and sloughing that usually attend it, with the possibility of septic absorption and consequent toxæmia, are elements requiring the most careful consideration. In a short time after the onset of phlegmonous erysipelas, the affected part becomes of a deep scarlet or dusky red hue, and very tense and firm to the touch. There is a sense of extreme heat, with quite severe pain, and from the first, symptoms of exhaustion may be present. If not interfered with, suppuration and sloughing usually occur, and may result in serious destruction of tissue. These results are dependent upon several conditions, viz., depreciation of the vitality of the part as a direct result of the intense inflammation, and extreme tension from inflammatory infiltration with the ensuing great disturbance of the circulation. The attendant exhaustion is in most cases directly proportionate to the amount of local destruction, hence the great importance of limiting it as far as possible. The first indication being to relieve the tension of the inflamed tissue, free incisions into the affected part must be made at once. We thereby secure a certain amount of local depletion in addition, which is highly beneficial. Some authorities recommend a single long incision, but this may give rise to considerable shock and hæmorrhage, and but imperfectly accomplish the desired object. It is therefore better to make a large number of small incisions, varying from one to three inches in length, the number and situation of which are to be regulated by the amount of tension present. The limb should now be fomented with hot carbolized or borated water until all hæmorrhage has ceased, and an "oakum poultice" applied. This consists in a thick layer of oakum or tow, saturated with water as hot as can be borne, and covered with a layer of oiled silk or muslin. The water should contain some antiseptic, and about ℨ ss. of the tincture of opium to the pint. These applications should be continued until resolution takes place, unless suppuration and sloughing occur in spite of prompt interference, when poultices of other kinds should be applied, to favor the separation of the sloughs. These may consist of the ordinary linseed meal, with the addition of laudanum, to increase the emollient effect, or if the sloughs and discharges be fetid, a poultice composed of equal parts of pulverized wood charcoal and linseed meal, mixed with brewer's yeast instead of water, should be used.

These disinfectant dressings lessen the liability to septic infection, and prevent fetor. All pocketing and collections of pus should be relieved by free incision. The importance of this cannot be over-estimated. The constitutional treatment is extremely important, and free stimulation should be the rule. Quinine should be given freely and preferably in small doses frequently repeated, a moderate degree of cinchonism being maintained. As a general rule, I administer about gr. ii. every two or three hours, unless gastric disturbance be thereby induced. The latter contingency is less liable to occur than when the drug is given in larger quantities, at longer intervals. The salicylate of iron should also be given in the doses before mentioned.

Milk punch is a useful form of stimulant and nourishment combined, and appears to be more acceptable to the stomach than egg-nog, which appears rather difficult of digestion. If obstinate vomiting occurs, the beef peptone will be found very serviceable, as it presents a greatly concentrated and readily assimilable form of nourishment. I prefer the preparation known as Gaunt's. This may be given in doses of a teaspoonful every hour, either in milk or a small quantity of water. If no gastric disturbance be present, the carbonate of ammonia and digitalis may be given, the character of the pulse being the criterion for its use. I

believe that this combination has a more markedly beneficial effect than would be accounted for by its action as a mere stimulant. When the stomach becomes absolutely intolerant of the various remedies, rectal alimentation and medication are, of course, necessary. The temperature, if excessive, should be controlled by the pack or cold sponging, as in the other forms of the disease. If the incisions be sufficiently numerous and free to relieve all tension, and are resorted to early in the disease, serious destruction of tissue rarely occurs, and consequently death from exhaustion is infrequent. Serious hæmorrhage is rare, it being usually readily controlled by styptics. In making the necessary incisions, care is to be taken not to allow the escape of a sufficient amount of blood to cause general depletion.

Cellulo-cutaneous and cellular erysipelas of the genital region are exceedingly dangerous, both from the especial tendency to asthenia, and the danger of extensive destruction of the parts involved, the cellular tissue in this situation being especially lax, and its vitality easily destroyed. The rapidity with which sloughing sometimes occurs, is astonishing, and I have seen the testes laid completely bare in a few days. In these cases, nothing but very radical measures are of any avail. A free crucial incision must be made for the whole extent of the swollen scrotum, with a sufficient number of smaller incisions upon both penis and scrotum, to relieve tbe tension. Hot antiseptic poultices should now be applied, and very free stimulation, with highly nourishing and concentrated diet resorted to. Large quantities of alcoholics, with the carbonate of ammonia and digitalis may be necessary. An ounce of brandy or whisky every hour may be beneficial in extreme cases. Although the destruction of tissue in some of these cases is very great, the amount and rapidity of repair is remarkable, as seen in the case I have cited, in which both testes were cleanly exposed, there being but a small ragged portion of integument left superiorly, but in which granulation and repair took place so perfectly that the organs were comfortably covered in, in the course of a few weeks. The scrotum was very greatly abbreviated, but no discomfort or pain were complained of by the patient.

There is little more to be said with especial reference to the cellular form of erysipelas, as the indications are essentially the same as in the severe cases of the other varieties. This type is marked by its still greater tendency to exhaustion, and the condition known as erysipelas typhoidea by some writers, and the more marked destruction of tissue, the inter-muscular areolar planes being often invaded, with the increased liability to septic absorption and resulting toxæmia. Especial care must consequently be taken, to permit the escape of pus and sloughs, with strict antisepsis, and an amount of stimulants regulated by the condition of the heart.

I wish incidentally to mention a peculiar affection, called by some superficial circumscribed gangrene, but which I think is really a form of erysipelas. This is usually met with upon the lower extremities, and in the few cases which have come under my observation, the disease began upon the dorsal or lateral surface of the foot, and extended upward upon the anterior surface of the leg. There is little pain or tumefaction of the part, which rapidly assumes a dusky, purplish-red hue, and soon becomes covered with large blebs, the surface beneath them being in some places like wash-leather or wet chamois skin. In a few days, the sloughing integument and cellular tissue separate and come away, and the tissues are laid bare down to the deep fascia and muscles. The affection is abruptly circumscribed, and does not seem to be connected with any special condition of constitutional depression. The treatment consists in tonics and hot poultices, and the cases result favorably if uncomplicated. Skin grafting is usually called for, when the ulcerations are in proper condition, thus preventing serious deformity from cicatricial contraction, which is apt to result. Some obscure local condition of enervation or vaso-motor disturbance may possibly be the prime factors in the pathology of these cases, but I think that they properly belong to the erysipelatous inflammations.

The special conditions existing in traumatic erysipelas require passing notice. They are an increased danger of septic infection, and in certain cases, the shock and anæmia resulting from the injury and its attendant hæmorrhage. Direct contagion is usually the source of these cases, and is rare, if antisepsis be properly attended to. Strict attention to cleanliness about the wound, and the liberal use of antiseptics, are indicated, as there is a danger of toxæmia proportionate to the extent of surface involved and capable of absorption.

In conclusion, I will endeavor to give a comprehensive *résume* of the treatment of erysipelas as I have presented it.

In the treatment of erysipelas, in all its forms, we have to consider:

First. The constitutional condition which may have predisposed to the disease.

Second. The lesion which has acted as its exciting cause, if the inflammation be traumatic.

Third. The tendency to local destruction of tissue, and the formation and burrowing of pus.

Fourth. The tendency to the typhoid condition, proportionate to the severity of the disease.

Fifth. The danger of septic absorption and toxæmia.

Sixth. The danger of propagation by contagion.

Seventh. The liability to serious deformity from cicatricial contraction.

1. The first indication is to be met by measures tending to correct the cachexia present, if possible. 2. The second, by careful attention to the wound, and rigid antisepsis. 3. The tendency to local destruction of tissue is to be combated by free incisions adequate to relieve all tension, and the application of hot dressings to maintain the vitality of the part and promote absorption of inflammatory exudate, and also to relieve the pain. The possibility of pocketing and burrowing of pus should be prevented by immediate incision into all purulent collections. 4. The supervention of typhoid symptoms should be prevented, if possible, by proper analeptic and tonic measures, and the liberal use of stimulants. 5. The fifth indication calls for perfect cleanliness and prompt removal of dead tissues which might excite decomposition of the discharges and consequent septic absorption; with the adoption of antiseptic dressing. 6. Transmission of the disease should be prevented by strict isolation of the affected, cleanliness, both in regard to the seat of disease and the instruments used, and on the part of the attendants, with the conscientious use of antiseptics. 7. Deformity may be prevented by careful management of the ulcers resulting from the disease, and the judicious use of skin grafting. Various mechanical contrivances may be necessary in certain situations, to prevent contraction.

137 *Madison St.*, *Chicago*, *June* 22, 1882.

